# The homing of bone marrow stem cells is differentially activated in ischemic and valvular heart diseases and influenced by beta-blockers

**DOI:** 10.1186/s12967-018-1520-9

**Published:** 2018-05-21

**Authors:** Melissa Kristocheck, Lucinara D. Dias, Carine Ghem, Bruna Eibel, Renato A. K. Kalil, Melissa M. Markoski

**Affiliations:** 1 Programa de Pós-Graduação em Ciências da Saúde–Cardiologia, Instituto de Cardiologia/Fundação Universitária de Cardiologia, Porto Alegre, RS Brazil; 2 Serviço de Patologia Clínica, Hospital de Clínicas de Porto Alegre, Universidade Federal do Rio Grande do Sul, Porto Alegre, RS Brazil; 3 Universidade Federal de Ciências da Saúde de Porto Alegre, Porto Alegre, RS Brazil; 4 Programa de Pós-Graduação em Ciências da Nutrição–UFCSPA, Rua Sarmento Leite, 245, Prédio III, Sala 507-Centro Histórico, Porto Alegre, RS CEP 90050-170 Brazil

**Keywords:** Cell homing, Stromal cell-derived factor-1, CXCR4, CXCR7, Ischemic heart disease, Valvular heart disease

## Abstract

**Background:**

Cell homing is the mechanism by which an injury releases signaling molecules that cause recruitment, proliferation, migration and differentiation of progenitor cells. Stromal derived factor-1 (SDF-1) and its receptor CXCR4 are key molecules involved in homing and little is known about their activation in cardiopathies. Here, we assessed the homing activation status of bone marrow cells (BMC) concerning the SDF-1 and CXCR4 expression in ischemic (IHD) and valvular (VHD) heart diseases.

**Methods:**

The SDF-1 and inflammatory profile were analyzed by ELISA from plasma obtained bone marrow of ischemic heart patients (IHD, n = 41), valvular heart patients (VHD, n = 30) and healthy controls (C, n = 9). Flow cytometry was used to evaluate CXCR4 (CD184) expression on the surface of bone marrow cells, and the CXCR4 expression was estimated by real-time quantitative PCR.

**Results:**

The SDF-1 levels in the groups IHD, VHD and control were, respectively, 230, 530 and 620 pg/mL (*P* = 0.483), and was decreased in VHD patients using beta-blockers (263 pg/mL) when compared with other (844 pg/mL) (*P* = 0.023). Compared with IHD, the VHD group showed higher CXCR4 (*P* = 0.071) and CXCR7 (*P* = 0.082) mRNA expression although no difference in the level of CXCR4^+^ bone marrow cells was found between groups (*P* = 0.360).

**Conclusion:**

In conclusion, pathophysiological differences between IHD and VHD can affect the molecules involved in the activation of homing. In addition, the use of beta-blockers appears to interfere in this mechanism, a fact that should be considered in protocols that use BMC.

## Background

Despite significant advances in therapeutic interventions, cardiovascular diseases (CVD) are still the leading cause of death worldwide. Among the CVD, ischemic heart disease (IHD) and non-ischemic valvular heart disease (VHD) are very frequent in older age. Despite significant improvement in the prognosis of these diseases, mortality rates remain high [1], mainly due to the limitation of conventional therapies to replace cardiomyocytes lost during cardiac ischemia [2].

Cellular cardiomyoplasty is an in situ implant of cells with the potential to induce cardiomyogenesis, angiogenesis and neovascularization, aiding the regeneration of damaged tissue [3]. In clinical practice, most studies have focused on the use of bone marrow-derived cells (BMC) in groups of patients with different heart diseases, describing a modest improvement in cardiac parameters, quality of life and patients’ symptoms [4]. Safety and effectiveness of therapy with BMC have also been demonstrated [5]. However, despite the well-designed, promising clinical studies, the mechanisms and molecules involved in cardiac repair and other issues that may optimize the beneficial effects of cardiomyoplasty remain to be clarified.

In cell therapy protocols, cell homing constitutes a critical step in the regeneration and repair process to promote cardioprotection and neoangiogenesis [6]. The level of expression of the chemokine SDF-1 and its receptor CXCR4 in stem cells influences the homing efficiency, so that the SDF-1/CXCR4 axis plays an essential role and offers potential advantages in cell therapy [7, 8]. Furthermore, CXCR7, another receptor with high affinity to SDF-1, is also involved in regenerating processes post-ischemic tissue [9].

Thus, considering the importance of cell homing to heart repair following injury, the present study aimed to assess the activation status of SDF-1/CXCR4 axes, in BM isolated from patients with IHD or VHD, target groups for stem cell therapy. We also analyzed the inflammatory profile and the influence of the use of some drugs, such as beta blockers, drugs widely used for these diseases, on the expression of the target molecules. Added to the existing knowledge about of stem cells homing, we can observe that the standard drug therapy used in cardiology, especially with beta-blockers, can interfere with the expression of molecules involved in this process, which should be considered in clinical trials on cell therapy for cardiology application.

## Methods

### Patients and design study

Patients aged between 40 and 70 years without hematologic diseases, cancer or chemotherapy treatment, were included in the study. Patients with history of acute myocardial infarction (AMI) and cardiac revascularization surgery were allocated to the IHD group (n = 41), and those with the diagnosis of aortic or mitral stenosis/insufficiency were allocated to the VHD group (n = 29). Both groups were using standard pharmacological therapy with different therapeutic classes used in cardiology (as acetylsalicylic acid, beta-blockers, statins, calcium-channels blockers, angiotensin-converting enzyme inhibitors, etc.). Previous to the median sternotomy, bone marrow (BM) aspirate samples were obtained from the sternum (20–30 mL). The surgeries were performed at the Instituto de Cardiologia/Fundação Universitária de Cardiologia, Brazil. The control group (C) was composed of healthy BM donors (n = 9), from whom samples were obtained from the iliac crest (20 mL).

### Immunophenotyping

The analysis of CXCR4^+^ cells was performed by immunophenotyping (n = 12) by flow cytometry. BM sample (2 mL) was incubated with lysing solution according to the manufacturer’s instructions (BD Biosciences, New Jersey, USA). Afterwards, the cells were incubated with 10 µL of PE-monoclonal antibody CD184 (BD Biosciences). The gate of lymphocytes and monocytes (including BMC), was determined based on side-scattered light (SSC) and forward-scattered light (FSC) parameters; at least 5000 events were acquired. The results are expressed as the percentage of stained cells (CD184^+^) inside the gate. Data acquisition and analysis were performed using a fluorescence activated cell analyser (FACSCanto II, BD) and the BD FACSDiva software.

### ELISA

Aliquots of 2 mL of BM sample were centrifuged at 1000 rpm for 10 min at 4° C, and plasma was stored at − 20° C until assayed. Plasma levels of SDF-1, isoform α (n = 79), and the inflammatory markers: interleukin-6 (IL-6, n = 40), interleukin-10 (IL-10, n = 40), and tumor necrosis factor-α (TNF-α, n = 40) were determined by the ELISA, with the aid of commercial kits (Quantikine; R&D Systems, Minneapolis, MN, USA) and Ebiosciences; RayBiotech, Norcross, GA, USA), and in accordance with the manufacturer’s instructions. All samples were measured in duplicate, by spectrophotometry (SpectraMax M2e, Molecular Devices, Sunnyvale, CA, USA), and data are expressed as picograms per milliliter (pg/mL).

### Real-time polymerase chain reaction (qRT-PCR)

Analysis of CXCR4 and CXCR7 gene expression was performed by qRT-PCR in samples collected from BM (IHD n = 11, VHD n = 11). Primers and probes for CXCR4, CXCR7 and GAPDH were obtained from the TaqMan Gene Expression Assay (Applied Biosystems, Waltham, MA, USA). GAPDH and healthy control samples were used as constitutive control and calibrator, respectively (n = 6). The expression levels of CXCR4 and CXCR7 cDNAs, synthesized from mRNAs isolated using the PureLink™ RNA Mini Kit Ambion (Life, Invitrogen, Carlsbad, CA, USA) were normalized to expression of the GAPDH. Changes in gene expression were determined by the equation 2^(−∆∆Ct)^ [10] and the results were transformed to logarithmic scale and expressed as relative levels in relation to the calibrator. Values greater than 1 were considered as increased expression level (upregulated) and those below 1, as decreased expression level (downregulated) in relation to the constitutive control.

### Statistical analysis

The data were expressed as mean and standard deviation or median and interquartile interval (25–75%). The Kruskal–Wallis and Student–Newman–Keuls tests were used to analyze the SDF-1 levels and other inflammatory markers. The Spearman’s or Pearson’s correlation coefficients were used to measure the associations between inflammatory markers and SDF-1 levels, and to determine the correlation between the variables obtained from cytometry, ELISA and quantitative PCR in control, IHD and VHD groups. For within-group comparison of gene expression, the Wilcoxon test followed by Kolmogorov/Smirnov test was used. Between-group comparisons of the presence of CXCR4^+^ cells and SDF-1 levels in relation to pharmacological therapy were performed by the Mann–Whitney test. Statistical significance was set at 5%.

## Results

### Patients’ baseline demographic and clinical characteristics

Study participants had a mean age of 58 years, and the majority of them (85%) were male. Clinical characteristics of patients were not different between the groups (Table 1). As expected, the presence of angina was more frequent in the IHD group than in VHD group (*P* = 0.008).

**Table 1:** Patients’ baseline demographic and clinical characteristic

Characteristics	IHD (n = 41)	VHD (n = 29)	*P*
Age (years)	57.95 ± 7.57	58.52 ± 11.09	0.800
Male (%)	35 (85.4)	20 (69.0)	0.177
Cardiovascular risk factors and comorbidities (%)			
Hypertension	35 (85.4)	22 (76.8)	0.487
Smoking	19 (46.3)	9 (31.0)	0.298
Diabetes mellitus	17 (41.4)	12 (41.3)	1.000
Dyslipidemia	13 (31.7)	5 (17.2)	0.277
Heart failure	6 (14.6)	8 (27.5)	0.302
Angina	21 (51.2)	5 (17.2)	0.008*
Coronary artery disease	10 (24.4)	3 (10.3)	0.239
LVEF (%)	63 ± 12	60 ± 14	0.839

Age (years) described in mean and standard deviation, *t* test. Other variables described by percentage (%), *Chi square test*. The control subjects (n = 9) had a mean age of 35 ± 15 years, which 22% were male and did not manifest the risk factors mentioned

*IHD* ischemic heart disease, *VHD* valvular heart disease, *LVEF* left ventricular ejection fraction

### CXCR4^+^ cells are present in same levels in the bone marrow of IHD and VHD patients

**Fig. 1:**
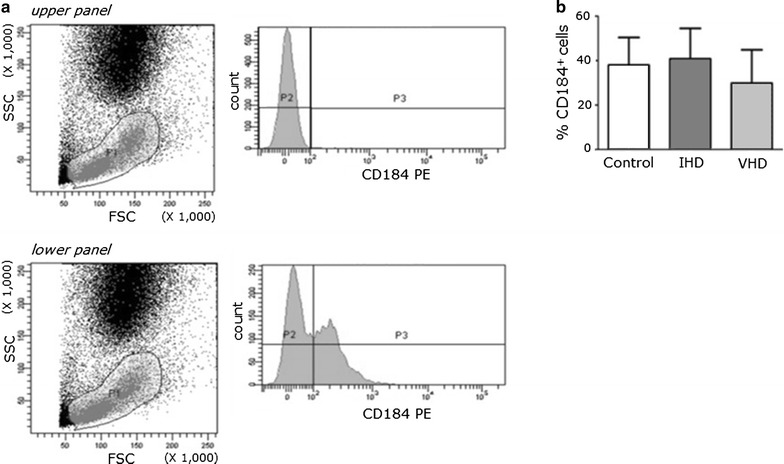
Analysis expression of CXCR4^+^ cells in the bone marrow. **a** Representative histogram of the flow cytometry; P1 sets the gate which includes BMC. Upper panel, sample not stained; lower panel, sample stained with antibody CD184-PE. **b** Percentage of CXCR4^+^ (CD 184-PE) cells in the population of BMC in groups of patients with ischemic heart disease (IHD), valvular heart disease (VHD) and healthy controls (Control)

The presence of CXCR4^+^ cells (CD184^+^) in the population of BMC in the groups of patients was investigated using flow cytometry. The CXCR4^+^ cells were characterized by expression of the marker CD184 (Fig. 1a). There was no difference between groups in the percentage of CXCR4^+^ cells (IHD: 38.8 ± 14.3 vs. VHD: 33.9 ± 9.4; *P* = 0.36) (Fig. 1b). In this experiment, the control group constitutes a representative sample (n = 3).

### Bone marrow of IHD patients showed lower levels of TNF-α but no differences for SDF-1 and other inflammatory markers in comparing to VHD patients

**Fig. 2:**
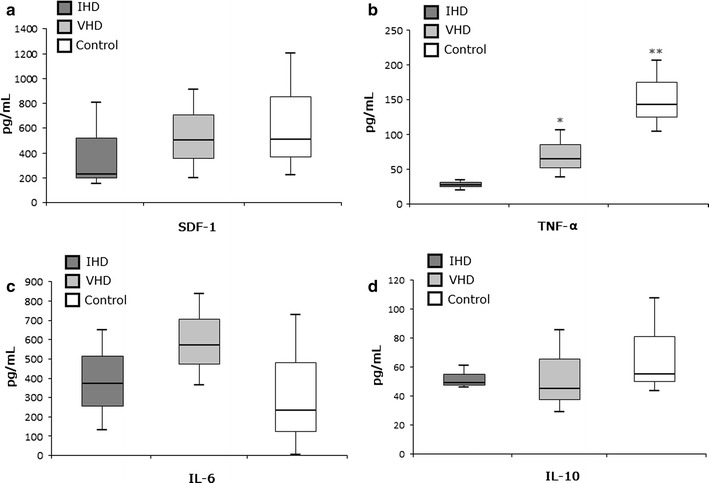
Analysis of SDF-1 levels and inflammatory cytokines. Values obtained by ELISA demonstrating the analysis of BM plasma levels expression of **a** SDF-1α; **b** TNF-α; **c** IL-6 and **d** IL-10 for ischemic heart disease (IHD), valvular heart disease (VHD) patients and healthy controls (Control). *IHD vs. VHD, P = 0.0112; **IHD vs. Control, P = 0.0003

Figure 2 shows the levels of cytokines measured from BM samples. SDF levels (Fig. 2a), even with reduced values in the IHD group (230.95 pg/mL) compared to VHD (501.34 pg/mL) and control (509.03 pg/mL) groups, showed no significant changes (*P* = 0.606 and *P* = 0.803, respectively). The TNF-α levels (Fig. 2b) were greatly reduced (*P* = 0.001) in IHD patients (28.12 pg/mL), both in relation to VHD (64.81 pg/mL) and the control (142.96 pg/mL, *P* = 0.003) participants. IL-6 and IL-10 levels (Fig. 2c, d) were, respectively, higher and reduced in BM samples from both cardiac patients in comparison to the control, but without statistical significance (*P* = 0.116 and *P* = 0.370, respectively). Additionally, IHD group showed a trend of association between the levels of SDF-1 and IL-6 (*r* = 0.461; *P* = 0.062) while VHD patients showed a trend of correlation for IL-6 and TNF-α (*r* = 0.447; *P* = 0.072).

### CXCR4 and CXCR7 mRNA are differentially expressed in bone marrow from IHD and VHD patients

**Fig. 3:**
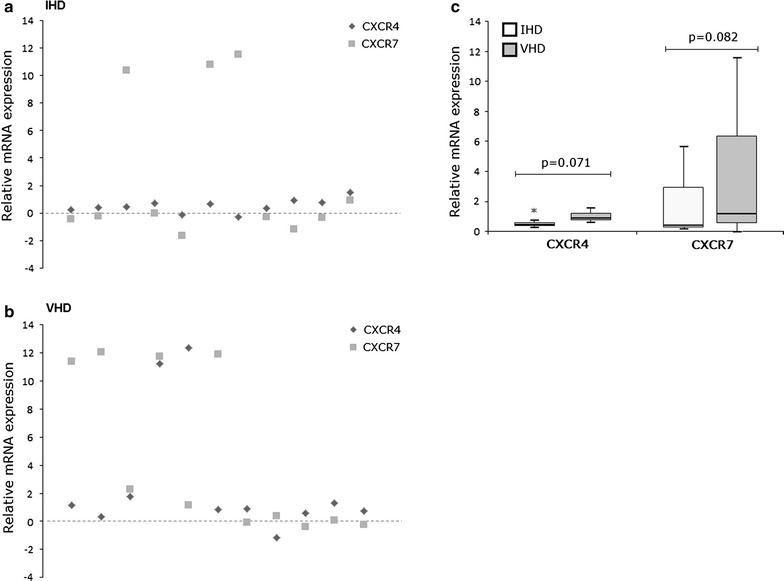
Representation of individual variations in the relative quantification of the expression of CXCR4 and CXCR7 genes obtained by qRT-PCR. **a** Individual expression levels for ischemic heart disease (IHD) and **b** valvular heart disease (VHD) patients (y axis = values of log_10_ 2^−∆∆Ct^/x axis = samples normalized by GAPDH and relative to the calibrator). **c** Relative expression of the mRNA of CXCR4 and CXCR7 in the IHD and VHD groups (median log_10_ 2^−∆∆Ct^). *CXCR4-IHD vs. CXCR7-IHD, P = 0.075

To visualize the gene expression profile of the SDF-1 receptors, CXCR4 and CXCR7, in BM samples and compare it between the IHD and VHD groups (Fig. 3), values of relative expression (2^−∆∆Ct^) by qRT-PCR were determined. Figure 3a shows individual analysis of the profile of receptor expression in the IHD group, demonstrating that the mRNA levels of CXCR4 were diminished compared to the constitutive control. Regarding the expression profile of the CXCR7 in the same group, the mRNA levels were increased in 3 samples in relation to GAPDH. In the VHD group (Fig. 3b), we found increased expression levels of CXCR4 mRNA in 5 samples, and increased expression of CXCR7 mRNA in 6 samples, as compared to GAPDH. Considering the mRNA expression comparison of receptors, we observed higher expression trend of CXCR4 (*P* = 0.071) and CXCR7 (*P* = 0.082) in VHD group than in IHD group (Fig. 3c). Within-group analysis showed no significant difference regarding gene expression levels of receptors. However, it was noted a tendency (*P* = 0.075) of higher CXCR4 expression in relation to CXCR7 in IHD patients, which was not observed for VHD (*P* = 0.236).

### VHD patients using beta-blockers showed lower levels of SDF-1

**Fig. 4:**
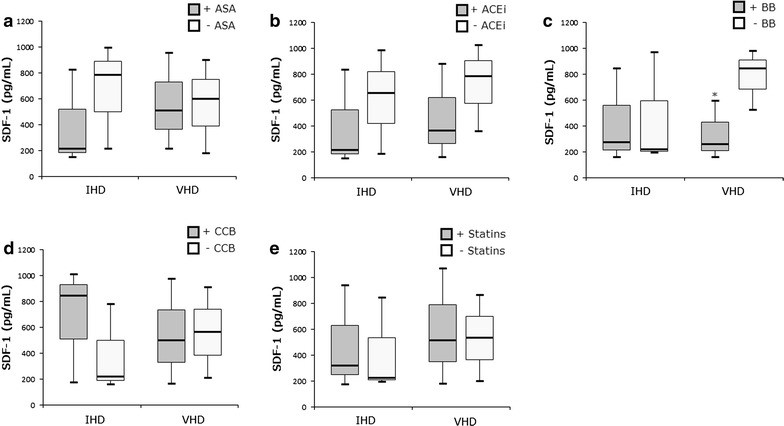
Influence of medications on the SDF-1 levels in cardiac patients. Ischemic heart disease (IHD) and valvular heart disease (VHD) patients had SDF-1 levels analyzed comparing the use (+) or not (−) of acetylsalicylic acid (ASA, **a**), angiotensin-converting enzyme inhibitors (ACEi, **b**), beta-blockers (BB, **c**), calcium-channels blockers (CCB, **d**), or statins (**e**). *VHD + BB vs. VHD − BB, *P* = 0.023

Since most of our patients were under continuous pharmacological treatment, we investigated its possible effect on SDF-1 levels (Fig. 4). VHD patients continuously using beta-blockers had lower SDF-1 expression levels (263 pg/mL) compared to those who did not use this drug (844 pg/mL) (*P* = 0.023) (Fig. 4c). The chemokine levels tended to be reduced for both diseases in patients using acetylsalicylic acid (Fig. 4a) or angiotensin-converting enzyme inhibitors (Fig. 4b), but without significant results (*P* = 0.099 and *P* = 0.126, respectively). We also did not observe a significant difference in the effects of calcium channel blockers (Fig. 4d) or statins (Fig. 4e) on SDF-1 levels (*P* = 0.264 and *P* = 0.427, respectively).

### VHD patients showed association of SDF-1 levels and CXCR4^+^ cells

Finally, we analyzed the degree of association between all the molecules tested and by different experimental approaches (SDF-1 levels, mRNA expression of CXCR4 and CXCR7, and CXCR4^+^ cells) for the samples of patients with IHD or VHD (Table 2). Accordingly, even with a small sample size, there was a positive strong correlation (*r* = 1, *P* < 0.05) between the presence of CXCR4^+^ cells and SDF-1 levels in the VHD group, within the bone marrow. This observation points that the homing process is enabled for these patients but may be harmed for the ischemic injury.

**Table 2:** Association of SDF-1 levels, CXCR4^+^ cells and mRNA expression of CXCR4 and CXCR7 receptors in the BM

Study variables	IHD	VHD
	r/*P*/n	r/*P*/n
SDF-1 vs. CXCR4^+^ cells	− 0.10/0.87/5	1.00*/< 0.05/4
SDF-1 vs. CXCR4 mRNA	0.60/0.20/6	− 0.21/0.62/7
SDF-1 vs. CXCR7 mRNA	− 0.71/0.11/6	0.64/0.11/7
CXCR4^+^ cells vs. CXCR4 mRNA	− 0.70/0.18/5	0.80/0.20/4
CXCR4^+^ cells vs. CXCR7 mRNA	0.00/1.00/5	0.40/0.60/7
CXCR4^+^ mRNA vs. CXCR7 mRNA	− 0.42/0.39/6	− 0.21/0.64/7

Stromal-derived-factor-1 (SDF-1, pg/mL); CXCR4^+^ populations (% CD184^+^ cells); CXCR4 and CXCR7 mRNA expressed in log10 of the relative expression

*r* Spearman’s correlation coefficient, *P* significance level, *n* sample, *IHD* ischemic heart disease, *VHD* valvular heart disease

* Strong direct correlation (P ≤ 0.05)

## Discussion

Many clinical studies of stem cell therapy in cardiology make use of BMC samples [4]. Issues related to molecular mechanisms and their relevance to the efficiency of cell therapy for heart repair have been rarely investigated. Considering the importance of stem cell homing to the cardiomyoplasty outcome [6], this study analyzed its activation status through the expression of key molecules involved in this mechanism, the chemokine SDF-1 and its receptor CXCR4 in BM samples of patients with ischemic and valvular heart diseases. The activation of this molecular axis is primordial to the start of the tissue repair process, such as what occurs after myocardial injury [11].

The activation and release of chemokines is a key mechanism in response to myocardial injury. In addition, there are multiple factors involved in homing of stem cells to heart diseases, which may in turn affect the production of chemokines in these conditions [12]. Among these, specific mention can be made to growth factors, as stem cell factor (SCF), and vascular endothelial growth factor (VEGF); cell adhesion molecules, as integrins, intercellular adhesion molecule-1 (ICAM-1), Monocyte Chemoattractant Protein-1 (MCP-1); and nitric oxide. SDF-1, particularly the α isoform, attracts stem cell to the lesion areas [7, 8], playing an essential role in protecting and repairing the ischemic organ. The increase of SDF-1 expression in response to a hypoxic environment [13], such as ischemia, acts as a cellular signal to attract CXCR4^+^ stem cells, which are potentially beneficial to cardiac repair [7]. Here, we evaluated plasma levels of SDF-1 in the BM of patients; and the data in literature reporting expression levels of this chemokine by BM are limited. However, even though showing reduction in cardiac patients, the chemokine levels did not differ significantly from healthy controls. Studies have shown controversial data regarding the levels of SDF-1 in pathological and physiological situations. Wyderka et al. showed decreased systemic levels of SDF-1 in ischemic patients when compared to those with valvular disease [14]. Chang et al. observed that serum levels of SDF-1 did not differ between AMI patients (1304 ± 591 pg/mL) and healthy control subjects (1264 ± 250 pg/mL) [15]. However, a recent study showed that patients with acute coronary syndrome (ACS) have high levels of systemic SDF-1 when compared to healthy control subjects. Furthermore, the authors identified SDF-1 as a potential biomarker for CVD in this cohort of patients [16]. Another key factor is the context of SDF-1 modulation, whether acute or chronic. Researchers found that the regulation of this chemokine is transient, with a significant increase in the acute phase and consequent decrease in the chronic phase of ischemia [17, 18]. This decline is due to the inactivation of SDF-1 by proteolytic enzymes and metalloproteinases [19].

Moreover, we could not evaluate the relationship between circulating and medullary concentrations of SDF-1. However, we believe that the SDF-1 values found in this study reflect the bioavailability of this molecule for tissue repair and regeneration, promoting the homing of circulating CXCR4^+^ cells. Inflammatory mediators, released in response to ischemic injury to limit tissue damage and create conditions for the start of the repair [12], may also have an effect on SDF-1 expression [20]. Here, we showed a trend in association between SDF-1 and IL-6 in IHD patients; both were slightly decreased in relation to the VHD group. Moreover, the levels of TNF-α were quite reduced in these patients. Thus, it is possible that, even within the BM, these molecules are necessary to trigger the homing, a process that may have been harmed in the ischemic patients.

Additionally, in our study, SDF-1 levels were decreased in VHD patients undergoing pharmacological therapy with beta-blockers compared to those who did not use medication. Zou et al. (2012) suggested that this pharmacologic class interferes in stem cell homing by decreasing SDF-1 and CXCR4 expression [21]. In fact, the IHD patients made more use of these drugs than the VHD and this may also be speculated as preventing the activation of homing.

Most of part of the biological effects of the chemokine SDF-1, including cardiac repair, is mediated by CXCR4/CD185 receptor [8]. For the interaction to occur, the receptor needs to be functionally active at the cell surface and coupled to G-protein [22]. Another receptor with high affinity to SDF-1 has been identified, the CXCR7 receptor. Studies suggest that CXCR7 perform as a supporting or receptor modulator to CXCR4 signaling and also be involved in the regulation of processes mediated by SDF-1 [9].

Evidence indicates that the levels of expression of CXCR4 on BMC surface determine the efficiency of homing [23]. In our study, there was no difference in the number of CXCR4^+^ BMC between the groups. By evaluating differences in the magnitude of CXCR4 and CXCR7 gene expression, we found high levels of mRNA of both receptors in the VHD group. Analyzing the expression of CXCR4 and CXCR7 to each patient individually, not necessarily the expression of two receivers was similar. Interestingly, we found that CXCR7 was higher for many patients, where the CXCR4 was low. Moreover, the increased CXCR4 mRNA expression was not accompanied by an increase in protein expression on the BMC surface. It should be observed that changes in mRNA expression levels do not necessarily reflect changes in protein expression levels, and therefore, it is important to determine the expression of the functional protein, which was a limitation of our study. Previous studies demonstrated that the expression of CXCR4 on the surface of the membrane is unstable, independent of the regulation of transcription, and effectively regulated by internalization receptor [24]. Thus, the intracellular storage of CXCR4 may at least partly explain the fact that increased mRNA expression of this receptor in the VHD group does not lead to an increase in CXCR4^+^ BMC.

Damas et al. observed in patients with angina, low levels of CXCR4 on the surface of peripheral blood mononuclear cells despite increased expression of the corresponding gene, as evidenced by mRNA levels in these cells [25]. In addition, the chronic phase of IHD may be related to low levels of gene expression of CXCR4 in this group of patients. Van Weel et al. have verified a decrease in mRNA expression of CXCR4 in the chronic phase of ischemia compared with the acute phase in patients with peripheral artery disease [26]. Thus, according to our data, factors such as SDF-1 levels, pharmacological treatment, and expression on the membrane surface may affect the expression and functionality of CXCR4, and consequently functional response of BMC. Efforts to mobilize the internalized receptor and increase the CXCR4^+^ cell expression [13, 27] may be of great value to reinforce the benefits of cell therapy for heart repair.

Despite a small sample size, we could demonstrate the expression profile of SDF-1, CXCR4 in a considerable number of cardiac patients, which allows us to infer the cell homing activation status of BMC in these patients. Considering that the chemoattractant function of SDF-1 correlates with the expression of CXCR4 on the cell surface [7], we found a strong direct correlation between CXCR4^+^ cells expression and SDF-1 plasma levels in the VHD group. This result is supported by the fact that these patients have shown higher SDF-1 levels as compared with the ischemic group. Then, one may assume that there may be a minimum level of the chemokine necessary to stimulate the activation of CXCR4^+^ cells in the BM. Additionally, it is worth mentioning the difference between bone marrow sources used in the study, since the control group samples obtained from the iliac crest, therefore for ethical reasons, it is unfeasible to collect MO sternum of healthy subjects, which is a limitation of the study. Issues such as the origin, regulation and impact of SDF-1 in circumstances that require tissue regeneration still remain incompletely understood. Finally, due to the difficulty in obtaining bone marrow samples not all study participants were evaluated for the expression profile of all molecules, a major limitation of this study.

Regarding the CXCR7 receptor, to date little is known about the function of this receptor during myocardial disease. In this study, it was observed a failure on the regulation of CXCR7 and CXCR4 receptors in both IHD and VHD groups. Some authors have hypothesized that the CXCR7 acts as a non-signaling receptor by removing extracellular SDF-1, and consequently, indirectly controlling the signaling of CXCR4 [28]. Studies reported that both the population of cells analyzed and the context (health/disease) may be considered contributing factors for the CXCR7 expression profile [29]. However, in view of the limitation of obtaining biological material of patients, we could not evaluate the expression of this receptor in the population of bone marrow cells evaluated in the study.

Our data demonstrate that the expression profile of key molecules involved in cell homing was altered in patients with heart disease. According to this profile and the central role of SDF-1/CXCR4 signaling in cell homing, our data show a trend towards decreased activation of this pathway in patients with IHD. Such functional impairment of BMC in cardiac patients may limit their therapeutic potential for clinical cell therapy. Factors such as pharmacotherapy, chronic phase of the disease, among others, could compromise the expression levels of these molecules, and hence the capacity to undergo cell therapy. The standard drug therapy in cardiology, especially with beta-blockers, deserves special attention because it can interfere with the expression of molecules involved in homing, jeopardizing this mechanism, a fact that should be considered in clinical trials on cell therapy, particularly for clinical application in cardiology. However, more studies are needed to establish the activation status of cell homing in the heart diseases, and the expression levels of molecules involved in this mechanism for therapeutic purposes.

## Conclusions

Pathophysiological differences between IHD and VHD can affect the molecules involved in the activation of homing. Here, expression the key molecules involved in activation of homing, as the chemokine SDF-1 and its receptor CXCR4 and CXCR7, showed variations once analyzed in BM samples of patients with ischemic and valvular heart diseases. We could verify that both cardiovascular diseases differentially affect the levels of these molecules, as well as, the inflammatory factors. In addition, the use of beta-blockers appears to interfere in this mechanism, a fact that should be considered in protocols that use BMC.

## Abbreviations

SDF-1: stromal derived factor-1
BMC: bone marrow cells
BM: bone marrow
CVD: cardiovascular diseases
IHD: ischemic heart disease
VHD: valvular heart disease
AMI: acute myocardial infarction
IL-6: interleukin-6
IL-10: interleukin-10
TNF-α: tumor necrosis factor-α
